# Monitoring cardiac symptoms during teleprehabilitation prior to coronary artery bypass grafting: a digital cardiac counselling trial subanalysis

**DOI:** 10.1093/ehjdh/ztag076

**Published:** 2026-05-19

**Authors:** Lieke van Susante, Rutger van Mierlo, Bart Scheenstra, Arnoud van ‘t Hof, Peyman Sardari Nia

**Affiliations:** Department of Cardiothoracic Surgery, Cardiovascular Research Institute Maastricht (CARIM), Maastricht University, Maastricht, Debyelaan 25, 6229 HX Maastricht, The Netherlands; Department of Cardiothoracic Surgery, Heart and Vascular Center, Maastricht University Medical Center, Maastricht, the Netherlands; Department of Cardiology, Heart and Vascular Center, Maastricht University Medical Center, Maastricht, the Netherlands; Department of Cardiology, Zuyderland Medical Center, Heerlen, the Netherlands; Department of Cardiac Rehabilitation, Basalt Rehabilitation, Leiden, the Netherlands; Department of Cardiology, Heart and Vascular Center, Maastricht University Medical Center, Maastricht, the Netherlands; Department of Cardiology, Zuyderland Medical Center, Heerlen, the Netherlands; Department of Cardiothoracic Surgery, Cardiovascular Research Institute Maastricht (CARIM), Maastricht University, Maastricht, Debyelaan 25, 6229 HX Maastricht, The Netherlands; Department of Cardiothoracic Surgery, Heart and Vascular Center, Maastricht University Medical Center, Maastricht, the Netherlands

**Keywords:** Teleprehabilitation, Cardiac surgery, Symptom burden, Preoperative assessment, Telemonitoring

## Abstract

**Aims:**

The purpose of this subanalysis was to explore whether or not teleprehabilitation leads to worsening cardiac symptoms in patients accepted for coronary artery bypass grafting (CABG).

**Methods and results:**

A subgroup of patients accepted for CABG (including off-pump and minimally invasive) who were randomized between teleprehabilitation and standard care in DCC trial were included. Teleprehabilitation patients had access to blended care supported by online modules, including nutritional support, functional exercise training, smoking cessation, inspiratory muscle training, and psychological support. All patients were weekly telemonitored for cardiac symptoms using a customized digital platform. This cohort included 155 patients. Baseline characteristics, including NYHA and CCS class, were balanced, except for shorter preoperative waiting times in weeks (15.0 [9.8–22.2] vs. 12.5 [7.0–16.0]) in the teleprehabilitation group. There was no significant difference in incidence of major adverse cardiac events in the preoperative period. There were 141 patients (77 control; 64 teleprehabilitation) with at least one symptom monitoring entry. A total of 373/1786 (21%) entries indicated worsening of cardiac symptoms. The distribution was right-skewed and did not significantly differ between the two groups. There was no statistically significant difference in time to event between control and teleprehabilitation for any cardiac symptom (*P* = 0.529), angina (*P* = 0.167), or dyspnoea (*P* = 0.334).

**Conclusion:**

Teleprehabilitation does not lead to worsening cardiac symptoms before CABG. We postulate that teleprehabilitation, which includes low-intensity physical training, can be safely offered to patients undergoing elective CABG.

## Graphical Abstract

**Graphical Abstract:**
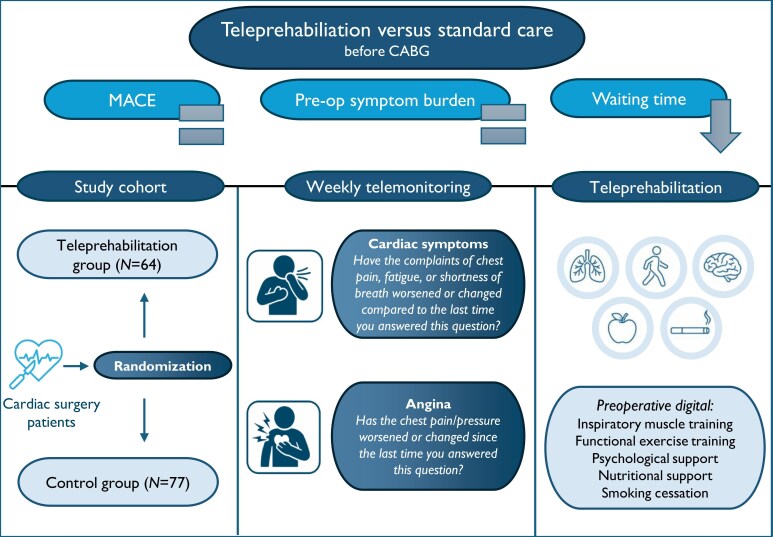

## Introduction

Teleprehabilitation enables both patients and clinicians to monitor cardiac symptoms remotely through telemonitoring. Teleprehabilitation appears to be a promising intervention, as it significantly reduces postoperative major adverse cardiovascular events and improves preoperative risk profiles.^1^ However, the effect of teleprehabilitation on preoperative symptom burden remains unclear. Teleprehabilitation requires physical and psychological exertion from patients; therefore, there is a possibility that it could worsen cardiac symptoms, which raises potential safety concerns.

The safety of prehabilitation has been established in cardiac surgery.^2,3^ These studies closely monitor patients and offer on-site supervision,^4^ which is in contrast to teleprehabilitation, in which patients are remotely monitored and supervised. Remote monitoring has the advantage that it informs clinicians about the stability of preoperative complaints over a longer period of time, which may offer an opportunity to re-evaluate the timing and indication of surgery. Telemonitoring has been similarly, and safely,^5^ implemented in the field of cardiac rehabilitation. Moreover, cardiac telerehabilitation has been accepted as an alternative to centre-based cardiac rehabilitation to improve participation and long-term adherence.^6^ Teleprehabilitation has a lot of overlap with cardiac telerehabilitation, whose recently published standards^7^ emphasize similar core components as teleprehabilitation, including remote risk factor management or exercise training counselling. Although telemonitoring is not yet a standard component of perioperative care guidelines in cardiac surgery, it is being recommended in these same guidelines.^8^

Increased physical and psychological activity could provoke cardiac symptoms, specifically in a remotely supervised environment. Since patients are remotely monitored through teleprehabilitation, one might question whether it could be safely implemented. A relevant discussion point, as previous research has shown that high preoperative symptom burden, like Canadian Cardiovascular Society (CCS) or New York Heart Association (NYHA) scores, results in poor clinical outcomes.^9–15^ Therefore, evaluating the effect of teleprehabilitation on symptom burden is highly relevant.

In our main analysis, teleprehabilitation reduced major adverse cardiovascular events (MACE) after elective cardiac surgery compared to standard care.^1^ This subanalysis assesses the effect of teleprehabilitation on self-reported cardiac symptoms among patients awaiting elective coronary artery bypass grafting (CABG). Second, we aim to explore what proportion of patients remain free of worsening cardiac symptoms before CABG. Our hypothesis is that teleprehabilitation does not significantly worsen cardiac symptoms in comparison to standard care. In addition, we expect a group of patients who remain free of worsening symptom burden while waiting for surgery. Therefore, we expect that teleprehabilitation can be safely implemented in standard cardiac surgery care.

## Methods

### Study design

This is a subanalysis of the DCC trial,^16^ a multicentre, randomized controlled trial conducted at Maastricht University Medical Center (MUMC+). Adult elective cardiac surgery patients were either randomized into the teleprehabilitation group, in which they received a personalized multimodal prehabilitation program for at least 6 weeks, or into the control group, in which they received regular pre-surgical care. This subgroup analysis included patients accepted for CABG (including Off-Pump Coronary Artery Bypass (OPCAB) and Minimally Invasive Direct Coronary Artery Bypass (MIDCAB)) without valve replacement. Randomization was done by stratification in blocks of four, six, or eight with random permutation. These strata were determined by the type of surgery and the EuroSCORE II. Patients lacking digital skills, experiencing Dutch language barriers, or predisposed to interfering medical conditions were deemed ineligible to participate in the study. All patients provided both written and telephone-based informed consent. The study was approved by the Medical Ethics Committee Maastricht in April 2020 (NL73754.068.20/METC20–028) and is registered on clinical trials.gov (NCT04393636). More detailed information about the design was published before.^16^

### Teleprehabilitation

All study participants were granted access to the customized Digital Cardiac Counselling platform, co-developed with Medify B.V. The platform contained audiovisual information regarding the care pathway and the surgical procedures. At baseline, all patients were screened for modifiable risk factors using a digital questionnaire available on the platform. Based on the screening results, participants in the teleprehabilitation group received tailored teleprehabilitation modules targeting their individual risk factors, with the aim of reducing their prevalence. The teleprehabilitation consisted of five modules: functional exercise training, inspiratory muscle training, nutritional support, psychological support, and smoking cessation. The modules were led by a multidisciplinary team experienced in cardiac rehabilitation, consisting of a cardiologist, nurses, physiotherapists, a psychologist, and dietitians. Patients received remote support through multiple video consultations with a member of the multidisciplinary team. In addition, educational materials related to their specific module were made available on the platform as part of a blended care approach.

### Symptom monitoring

All study patients were remotely monitored through the digital platform for symptomology. At study enrolment, patients were classified by their symptoms based on their NYHA functional class,^17^ and CCS class^18^ using a digital questionnaire. After this, patients received weekly notifications to evaluate their symptoms. Patients filled out two questions, asking them to compare any cardiac symptoms (angina, dyspnoea, and/or fatigue), and angina specifically to the week prior. These questions were:

Have the complaints of chest pain, fatigue, or shortness of breath worsened or changed compared to the last time you answered this question?

(Original Dutch translation: Zijn de klachten van pijn op de borst, vermoeidheid of kortademigheid verergerd of wisselend sinds de laatste keer dat u deze vraag beantwoordde?)

Has the chest pain/pressure worsened or changed since the last time you answered this question?

(Original Dutch translation: Is de pijn/druk op uw borst verergerd of wisselend sinds de laatste keer dat u deze vraag beantwoordde?)

These notifications continued until patients had undergone their surgery. Whenever symptoms worsened, the study team reached out to patients to take a history on these symptoms. If deemed necessary, patients were referred to their own cardiologist. Emergency services were contacted if symptoms were deemed severe or life-threatening.

### Data analysis

Baseline characteristics were presented for the teleprehabilitation group and control group. Normal distribution was tested for continuous variables using histograms, the Shapiro–Wilk test, and Kolmogorov–Smirnov test. Continuous variables were expressed as mean ± standard deviations, and categorical variables were shown as an absolute number (percentage) of the study population. We evaluated categorical baseline differences using the Pearson’s chi-squared test, and continuous variables with the independent Student’s *t*-test, or the non-parametric alternative when appropriate.

Patient characteristics included age, sex, body mass index (BMI), chronic obstructive pulmonary disease (COPD), diabetes mellitus (DM), smoking status, EuroSCORE II,^19^ socioeconomic status (SES),^20^ left ventricle ejection fraction (LVEF), estimated glomerular filtration rate (eGFR), New York Heart Association (NYHA) class, Canadian Cardiovascular Society (CCS) class, surgical approach (minimally invasive, conventional), surgery type (CABG, OPCAB or MIDCAB), treatment interval (<8 weeks, ≥8 weeks), length of hospital stay and pre-operative weeks. In this study, the preoperative period was defined as the number of weeks between trial enrolment, which started when informed consent was obtained after the multidisciplinary heart team had established the indication for CABG, and the week of surgery. This interval was considered the ‘’waiting time for surgery’’. All baseline characteristics of the participants were obtained from the hospital’s electronic health records, with the exception of the NYHA and CCS class, which were obtained from the Digital Cardiac Counselling platform, and the SES score, which were obtained through Statistics Netherlands. Statistics Netherlands assigns SES scores per neighbourhood, based on household data regarding welfare, educational level, and labour participation. Scores range from −1 (lowest SES) to +1 (highest SES), where 0 is the average SES in the Netherlands.

We evaluated symptom burden on multiple levels to gain a comprehensive understanding of its progression over time in both groups. Symptom burden was assessed for any cardiac symptom (angina, dyspnoea, and/or fatigue), as well as for angina alone. All patients were included in the baseline characteristics, including the patients who did not complete the monitoring questionnaire. However, these patients were not included in the main analysis regarding symptom burden, as it would be impossible to determine if patients had worsening symptoms in those weeks.

First, the total number of pre-operative weeks was calculated to determine how many opportunities patients had to fill out their symptom monitoring question, as well as how many times patients actually filled out the symptom monitoring questions. Then, we calculated the rate of symptoms by how many times patients filled out ‘’yes’’ in the symptom monitoring questions compared to the total number of instances they filled out either ‘’yes’’ or ‘’no’’. Second, we compared the incidence rate of worsening symptoms between the teleprehabilitation and control groups using negative binomial regression. This model accounted for differences in the number of possible instances of symptom worsening per patient by including the logarithm of exposure time as an offset. Results were expressed as incidence rate ratios (IRRs) with corresponding 95% confidence intervals and *P*-values. Third, the number of worsening weeks per patient was compared between the teleprehabilitation group and the control group using the Wilcoxon rank-sum test, and visualized in histograms. Between-group differences were quantified using Cliff’s delta to represent the magnitude and direction of the effect. Group medians and interquartile ranges (IQRs) were reported. Fourth, the time from enrolment to the first occurrence of cardiac symptoms, or angina, pre-surgery was analysed using Kaplan-Meier curves. Differences between the teleprehabilitation and control group were evaluated using the log-rank test, and Cox proportional hazards regression was used to estimate hazard ratios (HRs) and corresponding 95% confidence intervals (CIs). Last, we assessed the symptom burden during the last four weeks before surgery to discover whether patients go into surgery while experiencing worsening cardiac symptoms. Differences between the teleprehabilitation and control groups were analysed using the Chi-squared test or a non-parametric alternative, as appropriate.

In the main analysis, no differences were observed in the incidence of preoperative MACE. To gain a better understanding of the population included in this subanalysis, we evaluated the incidence of preoperative MACE specifically within this subgroup. Comparisons between the teleprehabilitation and control groups were performed using the Chi-squared test.

### Ethical considerations

The trial protocol was approved by the Medical Ethical Committee of Maastricht University Medical Center/Maastricht University (NL73754.068.20/METC20–028). The trial was independently monitored and audited by the Clinical Trial Center Maastricht, and performed in accordance with the principles of the Declaration of Helsinki. The authors assume responsibility for the accuracy and completeness of the data and analyses, as well as for the fidelity to the protocol.

## Results

### Baseline

**Figure 1:**
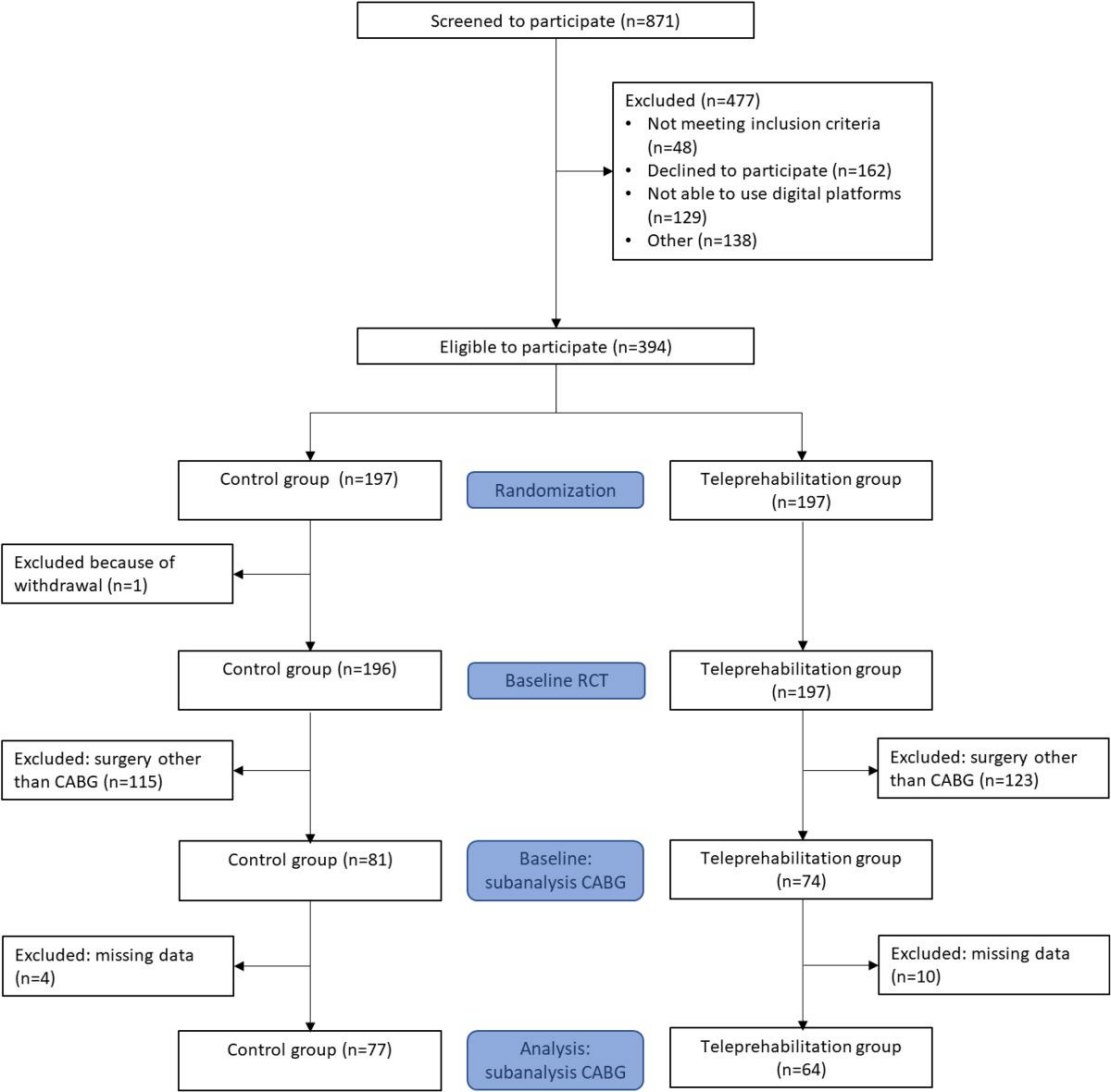
Flowchart of substudy.

This subanalysis included 155 patients; the mean age was 63.7 (± 8.3) years, and the majority of the patients were male [*n* = 132 (85%)]. In total, 74 patients were randomized to the prehabilitation group and 81 patients to the control group. There were 14 patients who did not fill out any symptom monitoring questionnaires, with a higher proportion in the teleprehabilitation group compared to the control group (*n* = 10 vs. *n* = 4; *P*  *<*  *0.001*); however, there were no baseline differences between these two groups, between responders vs. non-responders, or the incidence of MACE (*n* = 1 vs. *n* = 1). *Figure 1* shows the flowchart of this study. The baseline characteristics were balanced across both groups, except for fewer median preoperative weeks (prehabilitation: 15.0 [9.8, 22.2] vs. control: 12.5 [7.0, 16.0]) (*Table 1*).

**Table 1:** Baseline characteristics

	Control group (*n* = 81)	Teleprehabilitation group (*n* = 74)	*P*-value
**Demographic characteristics**			
Age, median [IQR]	63 [58.0–70.0]	65.0 [58.0–71.8]	0.291
Male, *n* (%)	67 (82.7)	65 (87.8)	0.503
**Health-related characteristics**			
BMI, kg/m^2^, median [IQR]	28.1 [25.4–32.2]	27.9 [24.5–30.0]	0.111
EuroSCORE II, median [IQR]	1.02 [0.77–1.38]	0.89 [0.64–1.37]	0.249
Diabetes mellitus, *n* (%)	24 (29.6)	21 (28.4)	1.000
Socio-economic status, mean ± SD	−0.05 ± 0.21	−0.03 ± 0.18	0.553
COPD, *n* (%)	7 (8.6)	5 (6.8)	0.890
Left ventricular ejection fraction, *n* (%)			0.648
<50	7 (8.6)	8 (10.8)	
≥ 50	74 (91.4)	66 (89.2)	
eGFR (mL/min/1.73 m^2^), median [IQR]	95.0 [74.0–119.0]	88.5 [71.0–105.8]	0.177
CCS class, *n* (%)			0.059
I	30 (37.0)	40 (54.1)	
II	36 (44.4)	29 (39.2)	
III	13 (16.0)	5 (6.8)	
IV	2 (2.5)	0 (0.0)	
NYHA class, *n* (%)			0.190
I	17 (21.0)	20 (27.0)	
II	30 (37.0)	30 (40.5)	
III	27 (33.3)	23 (31.1)	
IV	7 (8.6)	1 (1.4)	
**Surgery characteristics**			
Surgery type, *n* (%)			0.611
CABG	44 (54.3)	46 (62.2)	
MIDCAB	32 (39.5)	24 (32.4)	
OPCAB	5 (6.2)	4 (5.4)	
Surgical approach, *n* (%)			0.429
Minimally invasive	39 (48.1)	30 (40.5)	
Conventional	42 (51.9)	44 (59.5)	
Treatment interval, *n* (%)			0.429
<8 weeks	20 (25.0)	23 (31.1)	
>8 weeks	60 (75.0)	51 (68.9)	
Pre-op weeks, median [IQR]	15.0 [9.8–22.2]	12.5 [7.0 -16.0]	**0.033**
Length of hospital stay in days, median [IQR]	4.0 [3.0–5.0]	4.0 [3.0–5.0]	0.796

BMI, body mass index; CCS, Canadian Cardiovascular Society; CABG, Coronary Artery Bypass Graft; COPD, chronic obstructive pulmonary disease; eGFR, estimated glomerular filtration rate; IQR, interquartile range; LVEF, left ventricular ejection fraction; MACE, Major Adverse Cardiac Events; MIDCAB, Minimally Invasive Direct Coronary Artery Bypass; NYHA, New York Heart Association; OPCAB, Off-Pump Coronary Artery Bypass; SES, socioeconomic status.

### Cardiac symptoms

Of the 141 patients with at least one entry reported during the preoperative period, there were 2171 possible instances in which a response could be provided. Of these 2171 possible instances, 1786 entries were actually completed (82%) (*Table 2*). Among the completed entries, cardiac symptoms were reported 373 times (20.9%).

**Table 2:** Preoperative cardiac symptom burden

Group	Patients	Answers on symptom monitoring questions			Weeks with reported worsening (%)
		Yes	Yes or No	Possible instances	
**Angina, Dyspnoea, and/or Fatigue^a^**					
Control	77	222	1089	1330	20.4
Teleprehabilitation	64	151	697	841	21.7
Overall	141	373	1786	2171	20.9
**Angina** ^b^					
Control	77	174	1090	1330	16.0
Teleprehabilitation	64	100	697	841	14.3
Overall	141	274	1787	2171	15.3

^a^“Have the complaints of chest pain, fatigue, or shortness of breath worsened or changed compared to the last time you answered this question?”.

^b^“as the chest pain/pressure worsened or changed since the last time you answered this question?”.

In the control group, cardiac symptoms were reported in 222 of the 1089 completed entries (20.4%), and worsening angina was reported in 174 of the 1090 completed entries (16.0%).

In the teleprehabilitation group, cardiac symptoms were reported in 151 of the 697 completed entries (21.7%), and worsening angina was reported in 100 of the 697 completed entries (14.3%).

The data showed evidence of overdispersion for both the cardiac symptoms (Poisson dispersion = 3.786) and angina (Poisson dispersion = 4.898) outcomes, indicating that the negative binomial model provided a better fit (see Supplementary materials). For cardiac symptoms, the negative binomial model estimated an incidence rate ratio (IRR) of 0.89 (95% CI: 0.60–1.31, *P* = 0.560) in favour of the cardiac teleprehabilitation group. For angina alone, the negative binomial model yielded an IRR of 0.70 (95% CI: 0.42–1.17, *P* = 0.168), also in favour of cardiac teleprehabilitation. After correcting cardiac symptoms for baseline NYHA or CCS, there was a significant difference in favour of standard care in the Poisson model. However, this data showed evidence of overdispersion as well (Poisson dispersion = 3.170), which favours the fit of the negative binomial model that showed no statistical difference between groups (*P* = 0.763). Correcting for baseline CCS did not result in significant differences between groups for angina symptoms alone (negative binomial model: *P* = *0.594*).

**Figure 2:**
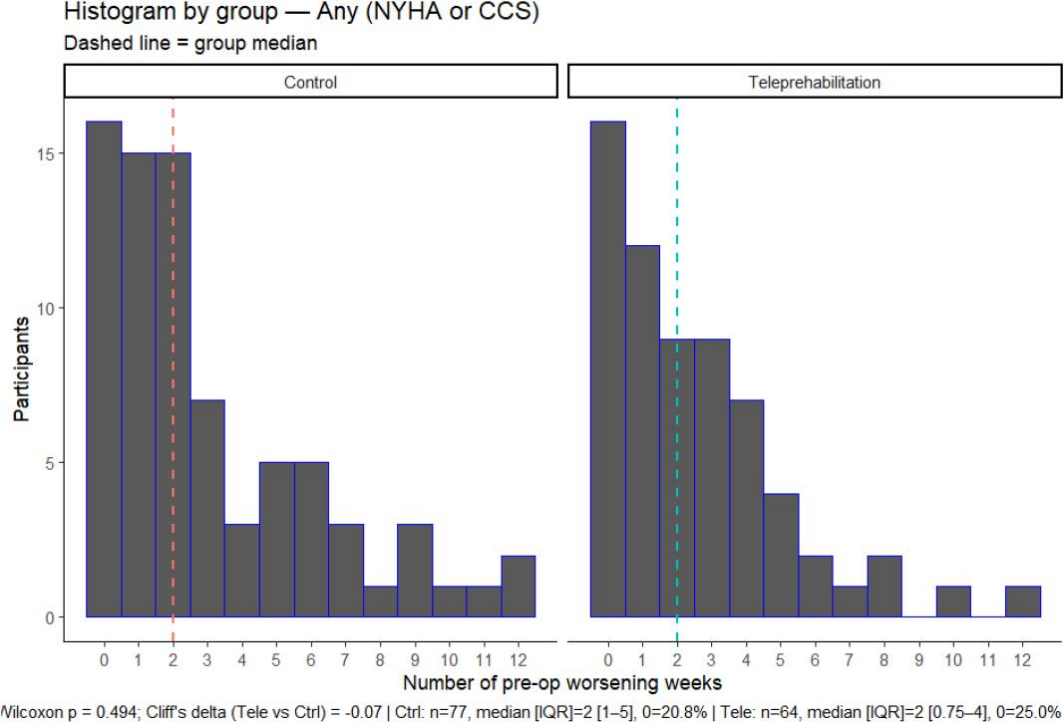
Distribution of the number of pre-surgical weeks with any worsening cardiac symptoms (angina, fatigue, or dyspnoea) within the teleprehabilitation and control group.

**Figure 3:**
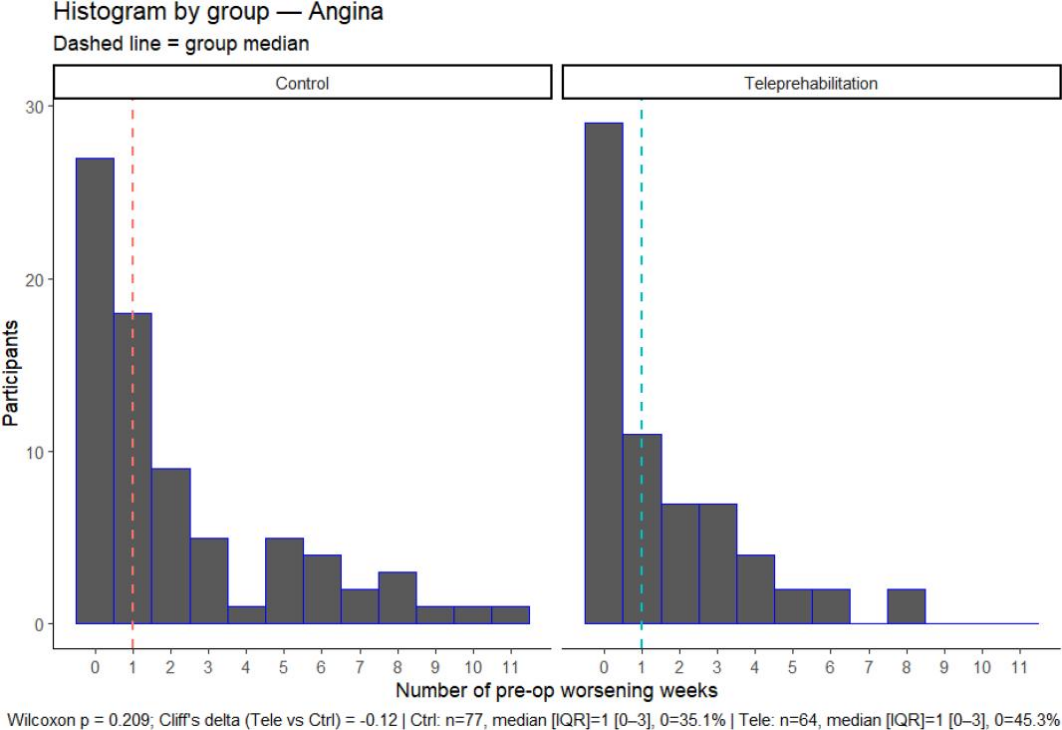
Distribution of the number of pre-surgical weeks with worsening angina within the teleprehabilitation and control group.

The histograms displaying the distribution of the number of preoperative weeks with worsening symptoms can be found in *Figures 2* and *3*. The distribution of pre-operative weeks of worsening symptoms was right skewed towards no worsening symptoms and not significantly different between the teleprehabilitation and control group for cardiac symptoms (Wilcoxon *P* = *0.404;* Cliff’s delta = −0.08)*, or* angina (Wilcoxon *P* = 0.209; Cliff’s delta = −0.12). The median number of preoperative weeks with worsening cardiac symptoms was 2 weeks in both groups, and for angina alone was 1 week in both groups.

**Figure 4:**
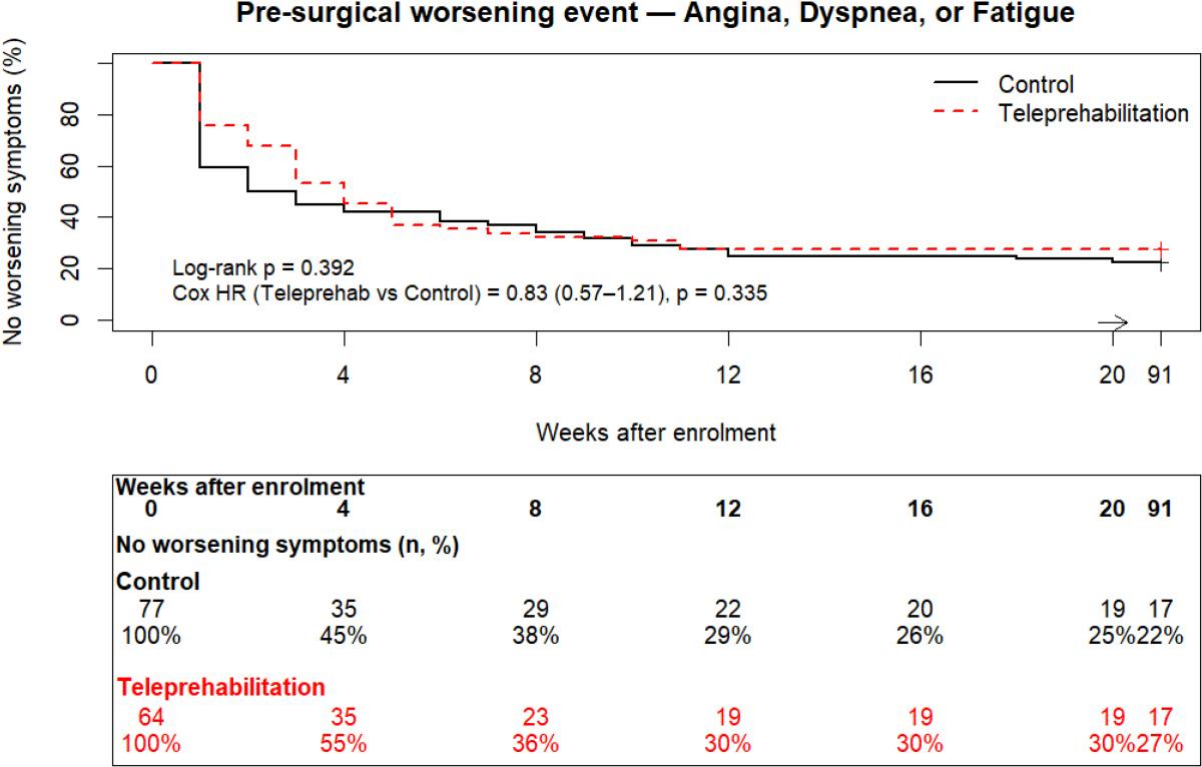
Time to first pre-surgical worsening of angina, dyspnoea, and/or fatigue.

**Figure 5:**
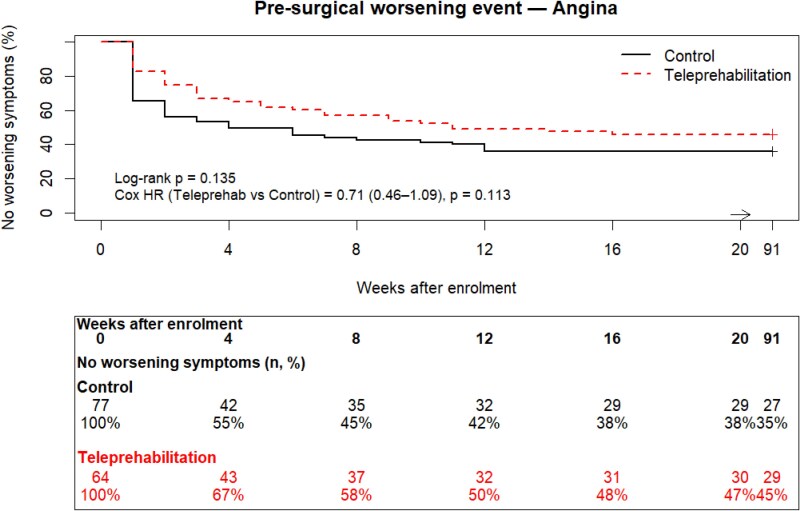
Time to first pre-surgical worsening of angina.

Time to first worsening cardiac symptoms is depicted in *Figures 4* and *5*. There was no statistically significant difference in time to event between control and teleprehabilitation for worsening angina, dyspnoea, and/or fatigue (log-rank *P* = 0.392; Cox HR = 0.83, 95% CI: 0.57–1.21, *P* = 0.335), or angina alone (log-rank *P* = 0.135; Cox HR = 0.71, 95% CI: 0.46–1.09, *P* = 0.113). The number of patients reporting at least one occurrence of worsening angina, dyspnoea, and/or fatigue was 107/141 (74%). Of the remaining 34 patients, 17/64 (27%) of the teleprehabilitation patients, and 17/77 (22%) of control patients reported no worsening symptoms (*P* = *0.535*). The occurrence of worsening angina symptoms was prevalent in 85/141 (60%) patients; 35/64 (55%) in the teleprehabilitation group, and 50/77 (65%) in the control group (*P* = *0.216*).

In the final four weeks before surgery, the number of patients filling out the symptom monitoring questions declined, and relatively fewer patients experienced cardiac symptoms, or angina alone (*Table 3*). Rates of cardiac symptom worsening ranged from 27% to 53% in the teleprehabilitation group and from 48% to 52% in the control group. Similar results were seen for angina alone, with proportions ranging from 23% to 38% in the teleprehabilitation group and 35% to 44% in the control group (*P* > 0.05 for all comparisons).

**Table 3:** Proportions of participants reporting worsening symptoms four weeks prior to surgery

Window	Control, a/N (%)	Teleprehabilitation, a/N (%)	Overall, a/N (%)	*P*-value
**Angina, Dyspnoea, and/or Fatigue**				
1 week	15/31 (48%)	6/22 (27%)	21/53 (40%)	0.206
2 weeks	20/41 (49%)	16/36 (44%)	36/77 (47%)	0.880
3 weeks	24/51 (47%)	21/45 (47%)	45/96 (47%)	1.000
4 weeks	28/54 (52%)	28/53 (53%)	56/107 (52%)	1.000
**Angina**				
1 week	11/31 (35%)	5/22 (23%)	16/53 (30%)	0.488
2 weeks	15/41 (37%)	13/36 (36%)	28/77 (36%)	1.000
3 weeks	20/51 (39%)	16/45 (36%)	36/96 (38%)	0.874
4 weeks	24/54 (44%)	20/53 (38%)	44/107 (41%)	0.611

### Incidence Major adverse cardiovascular events

In the teleprehabilitation group, two major adverse cardiac events were observed: one myocardial infarction and one earlier or repeated intervention (*Table 4*). In the control group, five major adverse cardiac events occurred, consisting of one myocardial infarction, two hospitalizations for heart failure or other life-threatening cardiac events, and two earlier or repeated interventions. The incidence of MACE did not differ significantly between groups (*P* = *0.446*).

**Table 4:** Incidence of Major adverse cardiovascular events in the pre-operative period in the teleprehabilitation and control group

	Control group (*n* = 81)	Teleprehabilitation group (*n* = 74)	*P*-value
Incidence preoperative MACE, *n* (%)	5 (6.2)	2 (2.7)	0.446
Cardiovascular death	0 (0.0)	0 (0.0)	
Myocardial infarction	1 (1.2)	1 (1.4)	
Stroke	0 (0.0)	0 (0.0)	
Hospitalization for heart failure or other life-threatening cardiac events	2 (2.5)	0 (0.0)	
Earlier or repeated intervention	2 (2.5)	1 (1.4)	

MACE, Major Adverse Cardiovascular Events.

## Discussion

Results from this subanalysis suggest that teleprehabilitation before CABG does not worsen cardiac symptoms, like angina, dyspnoea, and/or fatigue. Neither timing to the first instance of symptom worsening, nor symptom worsening in the last four weeks before surgery, was significantly affected by the participation in teleprehabilitation. Moreover, most patients in our trial did not experience frequent weeks of symptoms, with the majority experiencing fewer than 2 weeks of symptom worsening over the entire pre-surgical time period. Lastly, there were fewer major adverse cardiac events (MACE) in the preoperative period in the teleprehabilitation group than in the control group, which further supports its safety.

We believe that teleprehabilitation is safe, positively impacting surgical outcomes without worsening pre-operative cardiac symptom burden for patients.

Our findings are consistent with previous studies showing that daily physical exercise is well tolerated in patients with coronary artery disease. A previous randomized controlled trial in male patients has shown that exercise-based strategies outperformed percutaneous coronary intervention in stable disease, with superior event-free survival and exercise capacity.^21^ Despite this, patients are still frequently advised to limit physical activity before CABG in current clinical practice. Clinical decision making, for example, on the advice to limit physical activity before PCI or CABG, might benefit from understanding that invasive treatment by PCI or CABG is mechanistically different in acute and chronic CAD.^22^ Despite this evidence, concerns regarding the safety of teleprehabilitation persist, reflecting a gap between evidence and practice. Our findings address this gap and may contribute to a shift towards more proactive use of prehabilitation in patients awaiting CABG.

A notable finding in our study was that patients in the teleprehabilitation group had significantly shorter waiting times before undergoing CABG. This was unexpected, as all participants were enrolled at least eight weeks before surgery. This eight-week window allowed sufficient time for smokers to quit at least four weeks before surgery, thereby potentially reducing the risk of postoperative respiratory complications.^23,24^ One possible explanation for the reduced preoperative waiting time in our cohort of patients could be that the engagement in teleprehabilitation might make patients ‘ready’ for surgery more quickly than those without a structured prehabilitation plan. However, surgical scheduling for our cohort was determined independently of the research team, which would refute this argument, indicating that group allocation did not influence preoperative waiting times. We believe the observed difference in waiting time is most likely attributable to chance, given that this was a subanalysis of a larger randomized cohort. Furthermore, the trial was conducted during the COVID-19 pandemic, which, as in other hospitals, led to considerable rescheduling and overall increase of waiting time.^25^ Even though our control population experienced longer preoperative waiting times, the weekly symptom rate was no different from the teleprehabilitation population. As such, allowing a dedicated time period of, for example, eight weeks, to complete a telerehabilitation program should be feasible in regards to symptom burden.

Another notable finding in our study was the high rate of questionnaire completion amongst this cohort. This may, in part, be explained by a Hawthorne effect, whereby participants modify their behaviour simply because they are aware of being observed or monitored.^26,27^ Moreover, the proactive telephone follow-ups by researchers when patients reported worsening symptoms may have further motivated participants to complete their questionnaires consistently, as they recognized that their responses could directly influence subsequent contact or care. Evidence shows that, in general, patient activation leads to improved health outcomes and care experiences.^28^ Telemonitoring, particularly when integrated within a comprehensive teleprehabilitation program, may motivate patients to actively engage in their own healthcare, which could improve their physical, psychological, and functional readiness for elective CABG.

A major indication for CABG is symptom relief, which in some cases is the primary reason CABG is recommended by the multidisciplinary heart team (MHT). Deviation from the MHT recommendations is relatively rare, as shown by Pickering *et al.* (2025), in which adherence to the first multidisciplinary heart team recommendations were 96.4%.^29^ Striking is the rarity of deviating from the multidisciplinary heart team recommendation of CABG to Optimal Medical Therapy (OMT), which was only 3 out of 111 recommendations. A reason for deviating included symptom resolution.^30^ Our data suggest that there could be more patients with very low to no symptom burden. Accordingly, these patients could be re-assessed by the MHT to re-evaluate the initial recommendation for CABG. The re-assessment is a key step, since outright refusal of the recommendation of the MHT, without any re-assessment, results in poorer outcomes in MACE.^31^ Teleprehabilitation could be implemented to allow for improving the patients’ physical and mental health, and additionally to enable long-term symptom monitoring, which could support future reassessment of the indication for CABG. Importantly, previous research has shown a survival benefit for patients with multivessel disease and decreased LVEF undergoing CABG compared to PCI or OMT.^29^ Thus, any re-assessment could only take place for patients in whom the primary indication for CABG is symptom relief, not prognosis.

### Limitations

The results of the study cannot be extrapolated to patients with severe cardiac symptom burden (NYHA III-IV; CCS III-IV), as patients within these classes were poorly represented.

Furthermore, the findings cannot be applied to non-elective cardiac surgery patients, as only elective patients were included. More patients in the teleprehabilitation group did not fill out the symptom worsening questionnaires compared to those in the control group, resulting in more missing data. We believe that this difference in non-response might have been caused by the fact that the teleprehabilitation had regular contact with a healthcare professional via video consultations, which might have reduced their perceived need to fill out these questionnaires. A potential limitation is the observed trend towards lower baseline symptom severity in the teleprehabilitation group, suggesting a less symptomatic population study entry, which may have influenced the observed outcomes. However, even after accounting for these baseline differences, no clear advantage of either group was observed. This suggests that baseline differences in symptom severity are unlikely to have influenced the overall findings. Taken together, these results of the study best reflect a cohort of elective cardiac surgery patients with relatively low symptom burden. We are cautiously optimistic that a larger, powered cohort might reveal a benefit for low symptom burden participants. In this study, we evaluated symptom burden by asking patients to indicate whether symptoms had worsened compared to the week prior. A different, possibly more robust strategy is to ask the patients which CCS or NYHA stage they are in at the given week. Evaluating both current CCS and NYHA class, as well as symptom worsening would lead to more rich data, more easily translatable to clinical practice or research. Finally, a response bias should be considered. It is possible that patients experiencing more symptoms were more likely to complete the questionnaires, leading to an overrepresentation of symptom burden in the data compared to the actual prevalence.

### Future implications

So far, there are no randomized controlled trials specifically evaluating symptom burden before CABG, especially not in a teleprehabilitation group. Symptom burden might be important to track, since it provides more rich information on the patient’s clinical status. This might again be relevant for the MHT, as this could be a trigger for a re-assessment of the initial recommended treatment strategy, perhaps going for a less invasive approach for the time being.

## Conclusion

Teleprehabilitation does not worsen cardiac symptoms or increase the incidence of major adverse cardiac events before CABG. We postulate that teleprehabilitation, which includes low-intensity physical training, can be safely offered to patients undergoing CABG; however, further research is recommended. We consider teleprehabilitation a valuable addition to standard care, as it not only has the potential to reduce major adverse cardiac events but also provides an opportunity for telemonitoring. This approach allows systematically clinically stable, symptom-free patients to be reassessed within the multidisciplinary heart team.

## Lead author biography


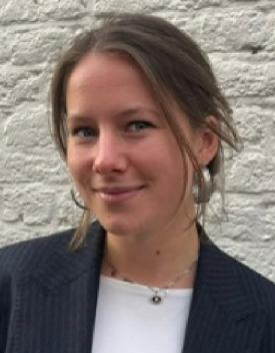


Lieke van Susante, MD, is a PhD candidate in cardiothoracic surgery at Maastricht University Medical Center. She obtained her medical degree from Maastricht University. Her research focuses on teleprehabilitation for patients undergoing cardiac surgery.

## Supplementary Material

ztag076_Supplementary_Data

## Data Availability

The data that support the findings of this study are available from the corresponding author upon reasonable request.
