# Supplementary material for: Monitoring cardiac symptoms during teleprehabilitation prior to coronary artery bypass grafting: a digital cardiac counselling trial subanalysis
Source: Eur Heart J Digit Health. 2026 May 19;7(5):ztag076. doi: 10.1093/ehjdh/ztag076 (PMC13228138; doi:10.1093/ehjdh/ztag076)
Supplement: ztag076_Supplementary_Data [file ztag076_supplementary_data.docx]

**Supplementary materials**

*Incidence rate ratios of pre-operative symptoms*

| **Model** | **IRR^a^** | **95% CI** | **P-value** | **AIC** | **Dispersion (Poisson)** |
| --- | --- | --- | --- | --- | --- |
| **Angina, Dyspnea, and/or Fatigue** | | | | | |
| Poisson | 1.063 | (0.86 - 1.31) | 0.564 | 791.905 | 3.786 |
| Negative Binomial | 0.891 | (0.60 - 1.31) | 0.560 | 616.520 | NA |
| **Angina** | | | | | |
| Poisson | 0.899 | (0.70 - 1.15) | 0.395 | 795.158 | 4.898 |
| Negative Binomial | 0.696 | (0.42 - 1.17) | 0.168 | 551.419 | NA |

*^a^ Teleprehabilitation compared to control. AIC = Akaike Information Criterion; IRR = Incidence rate ratio.*
